# A Review of Mammarenaviruses and Rodent Reservoirs in the Americas

**DOI:** 10.1007/s10393-022-01580-0

**Published:** 2022-03-05

**Authors:** Gloria Tapia-Ramírez, Consuelo Lorenzo, Darío Navarrete, Arturo Carrillo-Reyes, Óscar Retana, Rocío Carrasco-Hernández

**Affiliations:** 1 Departamento de Conservación de la Biodiversidad, El Colegio de La Frontera Sur, Periférico Sur S/N María Auxiliadora, 29290 San Cristóbal de Las Casas, Chiapas Mexico; 2 Departamento de Observación de la Tierra, Atmósfera y Océano, El Colegio de La Frontera Sur, Periférico Sur S/N María Auxiliadora, 29290 San Cristóbal de Las Casas, Chiapas Mexico; 3 Facultad de Ingeniería, Universidad de Ciencias y Artes de Chiapas, Av 1a. Sur Pte 1460, C.P., 29000 Tuxtla Gutiérrez, Chiapas Mexico; 4 Centro de Estudios en Desarrollo Sustentable, Universidad Autónoma de Campeche, Avenida Héroe de Nacozari 480, C.P., 24079 San Francisco de Campeche, Campeche Mexico; 5 Centro de Investigación en Enfermedades Infecciosas, Instituto Nacional de Enfermedades Respiratorias, Ismael Cosío Villegas, Calz. de Tlalpan 4502, C. P., 14080 Ciudad de México, Mexico

**Keywords:** Arenaviridae, *Mammarenavirus*, Rodents, Ecology, LUC, Viral hemorrhagic fever, Gloria Tapia-Ramírez

## Abstract

In the Americas, infectious viral diseases caused by viruses of the genus *Mammarenavirus* have been reported since the 1960s. Such diseases have commonly been associated with land use changes, which favor abundance of generalist rodent species. In the Americas—where the rates of land use change are among the highest worldwide—at least 1326 of all 2277 known rodent species have been reported. We conducted a literature review of studies between 1960 and 2020, to establish the current and historical knowledge about genotypes of mammarenaviruses and their rodent reservoirs in the Americas. Our overall goal was to show the importance of focusing research efforts on the American continent, since the conditions exist for future viral hemorrhagic fever (VHF) outbreaks caused by rodent-borne viruses, in turn, carried by widely distributed rodents. We found 47 species identified down to the species level, and one species identified only down to the genus level (*Oryzomys* sp.), reported in the Americas as reservoirs of mammarenaviruses, most these are ecological generalists. These species associate with 29 genotypes of *Mammarenavirus*, seven of which have been linked to VHFs in humans. We also highlight the need to monitor these species, in order to prevent viral disease outbreaks in the region.

## Introduction

Nearly 60% of infectious diseases in humans are zoonotic (Taylor et al. 2001; Jones et al. 2008). Zoonoses are diseases caused by pathogens, including bacteria, helminths, fungi and viruses, transmitted between animals and humans. Mammalian viruses are of special concern because some scholars have considered them potential agents for global pandemics (Jones et al. 2008; Geoghegan et al. 2016; Carrasco-Hernández et al. 2017).

According to Carlson et al (2019), 10,000 out of 40,000 viruses borne by mammals have zoonotic potential. The mammalian orders bearing the higher proportion of zoonotic viruses are bats, primates and rodents (Carlson et al. 2019). Specifically, rodents are reservoirs of 78 zoonotic viruses, belonging to the order Bunyavirales, comprised of species belonging to the Hantaviridae and Arenaviridae families (Laenen et al. 2019; Radoshitzky et al. 2019).

The Arenaviridae family consists of four genera: *Reptarenavirus*, *Hartmanivirus*, (both of them borne by reptiles)*, Antennavirus,* (borne by fishes) and, *Mammarenavirus*, borne by mammals, specifically rodents (Radoshitzky et al. 2019); although, there are reports of mammarenaviruses (Tacaribe virus) found in frugivorous bat *Artibeus lituratus* and in ixodid ticks (Downs et al. 1963; Sayler et al. 2014). The International Committee on Taxonomy of Viruses (ICTV, https://talk.ictvonline.org/taxonomy/) recognize forty genotypes of mammarenaviruses worldwide distributed, twenty-five are found in the Americas (Radoshitzky et al. 2019), seven of which are zoonotic agents of viral hemorrhagic fevers (VHFs) which could be severe or—sometimes—fatal for humans (CDC 2013).

Mammarenaviruses are bi-segmented RNA viruses with a genome of approximately 11,000 bp contained in two single-stranded segments of RNA (S and L): 3,400 bp in segment S, and 7,200 bp in segment L (Bausch and Mills 2014). In the Americas, mammarenaviruses are carried by rodents of the Cricetidae family, which consists of 765 species in 142 genera and 5 subfamilies (Pardiñas et al. 2017). The existence of rodent species reservoirs of mammarenaviruses have been reported in two subfamilies: Neotominae and Sigmodontinae (Sarute and Ross 2017).

Rodent reservoirs of mammarenaviruses have been described as generalists (Mills 2005) or having peridomestic habits (Charrel and de Lamberrie 2010). Their condition as generalists is important for viral transmission because it increases chances of contact between reservoirs and new hosts, promoting events of interspecies viral spillover (Charrel and de Lamberrie 2010). Their role as reservoirs is favored by their morphological, physiological, and behavioral characteristics; for example, they have a high reproductive potential and reach high population densities, which contributes to viral transmission (Mills et al. 2007).

Worldwide, there is evidence on the role of rodents in the emergence of VHFs caused by mammarenaviruses. In the Americas, the first VHF was reported in Argentina in 1955 (Arribalzaga 1955) where the responsible rodent transmitting the virus to humans was *Calomys musculinus.* Two other VHFs were later reported in South America, in Bolivia in 1959 (Mackenzie et al. 1964) and Venezuela in 1989 (Salas et al. 1991). Involved rodent species were *Calomys callosus* and *Zygodontomys brevicauda,* respectively. In Mexico, an epidemic outbreak in the state of Chiapas was reported in 1967 (Goldsmith and Shields 1971), with symptoms similar to those of South American VHFs. The rodent species involved was *Peromyscus mexicanus*. All aforementioned rodent species have the following characteristics in common: they are widely distributed, have a high reproductive rate and the ability to colonize a wide variety of habitats (Sarute and Ross 2017). The VHFs present in the Americas can reach human mortality rates between 5–30% (PAHO 2021).

After the first outbreak in Argentina, some light was shed on the main drivers of VHFs emergence. Land use change (LUC) was considered the main driver in the emergence (Mills et al. 1992; Charrel and de Lamberrie 2010), because it promotes habitat destruction and changes in the original landscape structure; which modifies community rodent structure (Suzán et al. 2008; García-Peña et al. 2021), then, leading to greater numbers of generalists than specialists (Murphy and Romanuk 2014). Generalist species have the ability to rapidly colonize more than one habitat type and achieving high population densities, thereby favoring epidemic outbreaks (Mills 2005; Sarute and Ross 2017).

Since the Americas are under an accelerated LUC process and neglected diseases occur—caused by rodent borne mammarenaviruses—the objective of the present study was to review and compile information published in digital media regarding the role of native rodents as reservoirs of *Mammarenavirus* in the Americas. Our overall goal was to show the importance of focusing research efforts on the American continent, since the conditions exist for future outbreaks of VHFs caused by rodent borne viruses of wide distribution.

Therefore, we compiled information bringing together up-to-date data on genotypes of mammarenaviruses and their rodent reservoirs. Likewise, we show areas of confluence of more than one species of reservoir rodent on the continent. We have synthesized the available data on the relationship between land use change and the emergence of viral hemorrhagic fevers, and how this relationship is linked to the species’ ecology and it is shaped by environmental factors. Finally, data on the phylogenetic and evolutionary relationships between mamarenaviruses and their rodent reservoirs were gathered.

## Methods

Using the search engines PubMed, Web of Science and Google Scholar we carried out a search of scientific articles published from 1960 to 2020 containing any combination of the following key words in the title or in the abstract: *Arenavirus*, Arenaviridae, *Mammarenavirus*, rodent reservoir, and the Americas. Year interval attending to the first outbreak reported in the Americas, in Argentina, near 1960. We also carried out a search of all arenaviruses recognized by the International Virus Taxonomy Committee (Radoshitzky et al. 2019).

Additionally, using the IUCN platform (IUCN 2020), we obtained geographic distributions for all reservoir species reported in the present document. We then identified overlapping areas of these distributions to visualize areas of convergence of more than one rodent reservoir species, where it could be necessary to put efforts for future studies because the potential contact between people and rodents. IUCN geographic distributions are accessible data, curated by experts in each species and are available for all the rodent species in this study. Previously have been used to show the overlapping promotes the co-circulation of viruses in the Americas (Luis et al. 2015; Milholland et al. 2018; Shipley et al. 2019).

Finally, we generated a series of maps containing all the above-mentioned information using QGIS 3.10.14 (QGIS Development Team 2020).

With the available data on LUC, along with the ecological and environmental factors, a conceptual framework was built explaining the role that LUC plays in the emergence of viral hemorrhagic fevers on the continent; as well as how some ecological and environmental factors interact to shape the response of rodents to these changes. Additionally, we try to explain the transmission routes of mammarenaviruses among the rodent populations and between species. The present study incorporates data from the southern United States to central Argentina.

## Results

Our literature search generated 245 articles. In the first filter, all the reviews and chapter books were dismissed (28 items), because they were a compilation of previously published information and we were on the search of recent field publications. A second filter included reading the abstract of 217 articles, looking for the words of the search criteria described above. From 217 articles, 156 did not fulfill search criteria, that is, even though the arenaviridae was their main topic, there was no mention about rodent reservoirs or they were articles about *Mammarenavirus* of the Old World, with just a brief mention to the New World species; thus they were excluded. Finally, 61 articles were selected which had been published in 29 indexed journals.

### Rodents Reservoirs and Mammarenaviruses

In the Americas, there are 47 species identified down to the species level, and one species identified only down to the genus level (*Oryzomys* sp.), of rodent reservoirs of mammarenaviruses. They are currently known to carry 27 of the 29 genotypes of *Mammarenavirus*. The remaining two genotypes have no rodent reservoir assigned yet (Table 1). Twenty-five genotypes are recognized by the International Committee on Taxonomy of Viruses (ICTV). There is a *Mammarenavirus* in the Americas not bearing by rodents but by bats, Tacaribe virus (TCV), included in Table 1 for the record.

**Table 1:** Mammal reservoir species of genotypes of *Mammarenavirus* in the Americas.

Reservoir species	Habitat type	*Mammarenavirus* species (according to ICTV)	Virus name (Abbreviation)	Country occurrence of virus	Disease reported	References
Order Rodentia						
Family Cricetidae						
Subfamily Netominae						
*Neotoma albigula*	Desert, rocky areas	*Whitewater Arroyo mammarenavirus*	Whitewater Arroyo virus (WWAV)	US	Fatal illnesses associated	Fulhorst et al. (1996)
			Big Brushy Tank virus (BBTV)	US	NR	Milazzo et al. (2008)
			Tonto Creek virus (TTCV)	US	NR	Milazzo et al. (2008)
*Neotoma leucodon*	Shrubland, rocky areas, desert	NSND	*Real de Catorce virus (RCTV)	MEX	NR	Inizan et al. (2010)
*Neotoma macrotis*	Desert, shrubland, forest	*Bear Canyon mammarenavirus*	Bear Canyon virus (BCNV)	US	NR	Fulhorst et al. (2002)
*Neotoma mexicana*	Pine-oak forest	*Whitewater Arroyo mammarenavirus*	Skinner Tank virus (SKTV)	US	NR	Cajimat et al. (2008)
*Neotoma micropus*	Shrubland		Catarina virus (CTNV)	US	NR	Cajimat et al. (2007)
*Oryzomys palustris*	Wetland, grassland	*Tamiami mammarenavirus*	Tamiami virus (TAMV)	US	NR	Calisher et al. (1970)
*Peromyscus californicus*	Coniferous and oak woodland	*Bear Canyon mammarenavirus*	Bear Canyon virus (BCNV)	US	NR	Fulhorst et al. (2002)
*Peromyscus mexicanus*	Semideciduous secondary forest, coffee groves, arable land	NSND	*Ocozocoautla de Espinosa virus (OCEV)	MEX	NR	Cajimat et al. (2012)
Subfamily Sigmodontinae						
*Akodon azarae*	Scrub meadows, wetland, shrubland	*Argentinian mammarenavirus*	Junin virus (JUNV)	ARG	Argentine Hemorrhagic Fever	Parodi et al. (1958)
*Calomys laucha*	Forest, grassland, arable land					
*Calomys musculinus*	Shrubland, pastureland, arable land					
*Calomys callosus*	Shrubland, pastureland, arable land, rural gardens, heavily degraded forests	*Machupo mammarenavirus*	Machupo virus (MACV)	BOL	Bolivian Hemorrhagic Fever	Johnson et al. (1963)
		*Latino mammarenavirus*	Latino virus (LATV)	BOL	NR	Webb et al. (1973)
*Calomys callidus*	Shrubland		Latino virus (LATV)	BRA		Fernandes et al. (2015)
*Calomys tener*	Shrubland, grassland, pastureland, arable land, urban areas, heavily degraded forests	NSND	*Pinhal virus (PINV)	BRA	NR	Bisordi et al. (2015)
*Hylaeamys megacephalus*	Primary, secondary, and degraded forests	*Cupixi mammarenavirus*	Cupixi virus (CPXV)	BRA	NR	Charrel et al. (2002)
*Neacomys guianae*	Forest subtropical	*Serra do Navio mammarenavirus*	Amaparí virus (AMAV)	BRA	NR	Pinheiro et al. (1977)
*Neacomys musseri*	Tropical forests	*Xapuri mammarenavirus*	Xapuri virus (XAPV)	BRA	NR	Fernandes et al. (2018)
*Necromys benefactus*	Forests, savanna, grassland, heavily degraded forests	*Oliveros mammarenavirus*	Oliveros virus (OLVV)	ARG	NR	Bowen et al. (1996)
*Necromys lasiurus*	Forests, savanna, grassland, heavily degraded forests		Oliveros virus (OLVV)	BRA	NR	Fernandes et al. (2015)
*Nephelomys albigularis*	Tropical forests	*Cali mammarenavirus*	Pichindé virus (PICV)	COL	NR	Trapido and Sanmartin, (1971)
*Oecomys bicolor*	Tropical forests	*Allpahuayo mammarenavirus*	Allpahuayo virus (ALLV)	PER	NR	Moncayo et al. (2001)
*Oecomys paricola*	Tropical forests					
*Oecomys* sp.	Tropical forests	NSND	*Patawa virus	FGU	NR	Lavergne et al. (2015)
*Oligoryzomys fornesi*	Gallery forests, shrublands, grasslands	*Planalto mammarenavirus*	Aporé virus (APOV)	BRA	NR	Radoshitzky et al. (2015)
*Oryzomys* sp.	No information	*Flexal mammarenavirus*	Flexal virus (FLEV)	BRA	Febrile Illness in laboratorists	Pinheiro et al. (1977)
*Sooretamys angouya*	Tropical forests, savana, heavily degraded forests	*Paraguayan mammarenavirus*	Paraná virus (PARV)	PAR	NR	Webb et al. (1970)
*Sigmodon alstoni*	Shrublands, grasslands	*Guanarito mammarenavirus*	Guanarito virus (GTOV)	VEN	Venezuelan Hemorrhagic Fever	Salas et al. (1991)
		*Pirital mammarenavirus*	Pirital virus (PIRV)	VEN	NR	Fulhorst et al. (1997)
*Zygodontomys brevicauda*	Tropical forests, savanna, marshes, arable lands	*Guanarito mammarenavirus*	Guanarito virus (GTOV)	VEN	Venezuelan Hemorrhagic Fever	Salas et al. (1991)
Unknown	No information	*Chapare mammarenavirus*	Chapare virus (CHPV)	BOL	Bolivian Hemorrhagic Fever	Delgado et al. (2008)
Unknown	No information	*Brazilian mammarenavirus*	Sabiá virus (SABV)	BRA	Hemorrhagic Fever	Coimbra et al. (1994)
Family Muridae						
Subfamily Murinae						
*Mus musculus*	Urban	*Lymphocytic choriomeningitis mammarenavirus*	Lymphocytic Choriomeningitis virus (LMCV)	US, ARG, COL & FGU	Febrile illness	Childs et al. 1992; Riera et al. 2005; Lavergne et al. 2016; Castellar et al. 2017)
Order Chiroptera						
Family Phyllostomidae						
*Artibeus lituratus*	Forests	*Tacaribe mammarenavirus*	Tacaribe virus (TCRV)	TRI	NR	Downs et al. (1963)

^*^Mammarenavirus genotypes which are not yet recognized by The *International Committee in Taxonomy of Virus* (ICTV. Abbreviations: *Ref* Reference, *NSND* No Species Name Designated, *NR* Not Registered, ARG Argentina, BOL Bolivia, BRA Brazil, COL Colombia, FGU French Guiana, MEX Mexico, PAR Paraguay, PER Peru, TRI Trinidad, US United States, VEN Venezuela.

**Figure 1:**
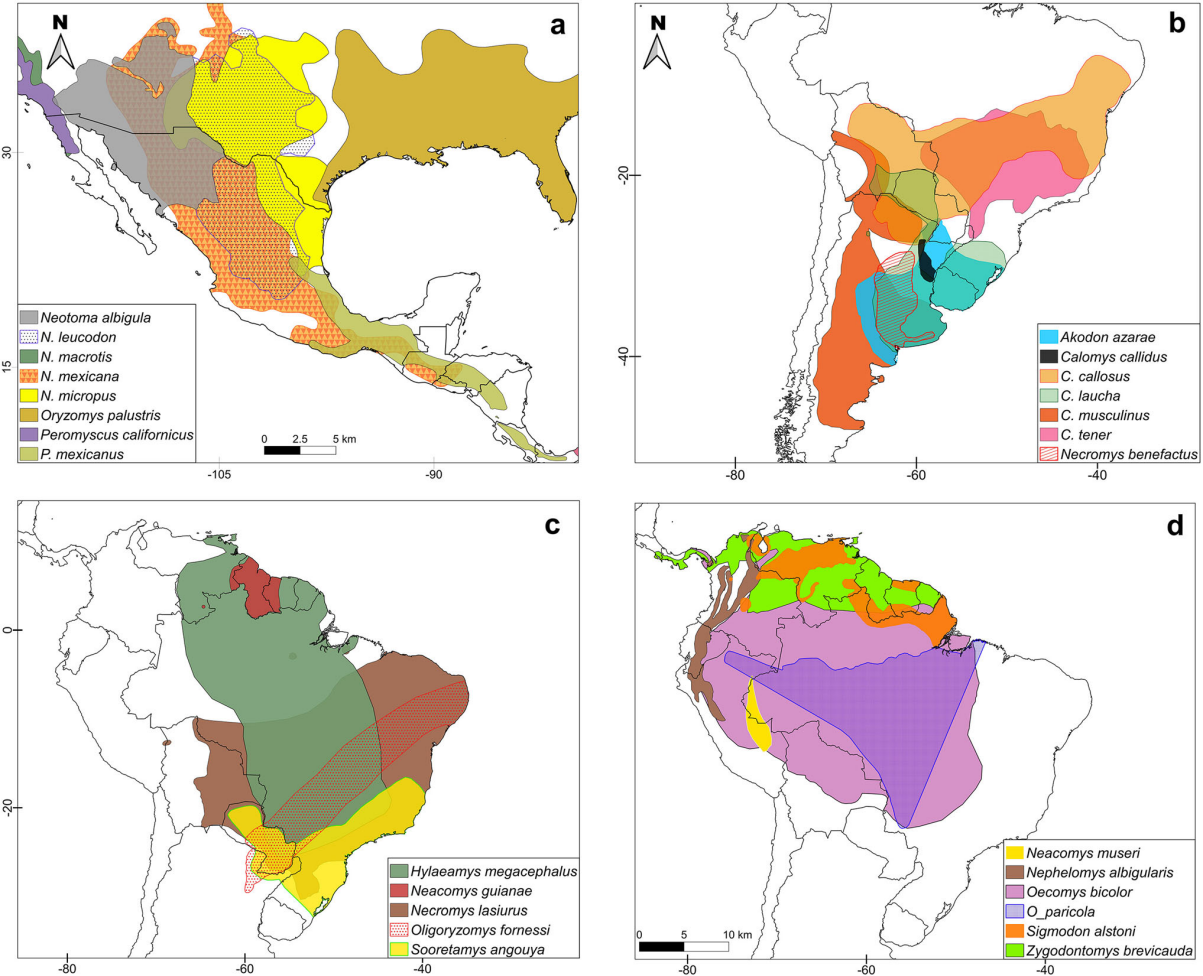
Geographic distributions of rodent reservoir species of mammarenaviruses in the Americas: **a** North America, **b**, **c** & **d** South America Source of data distributions: IUCN (2020).

**Figure 2:**
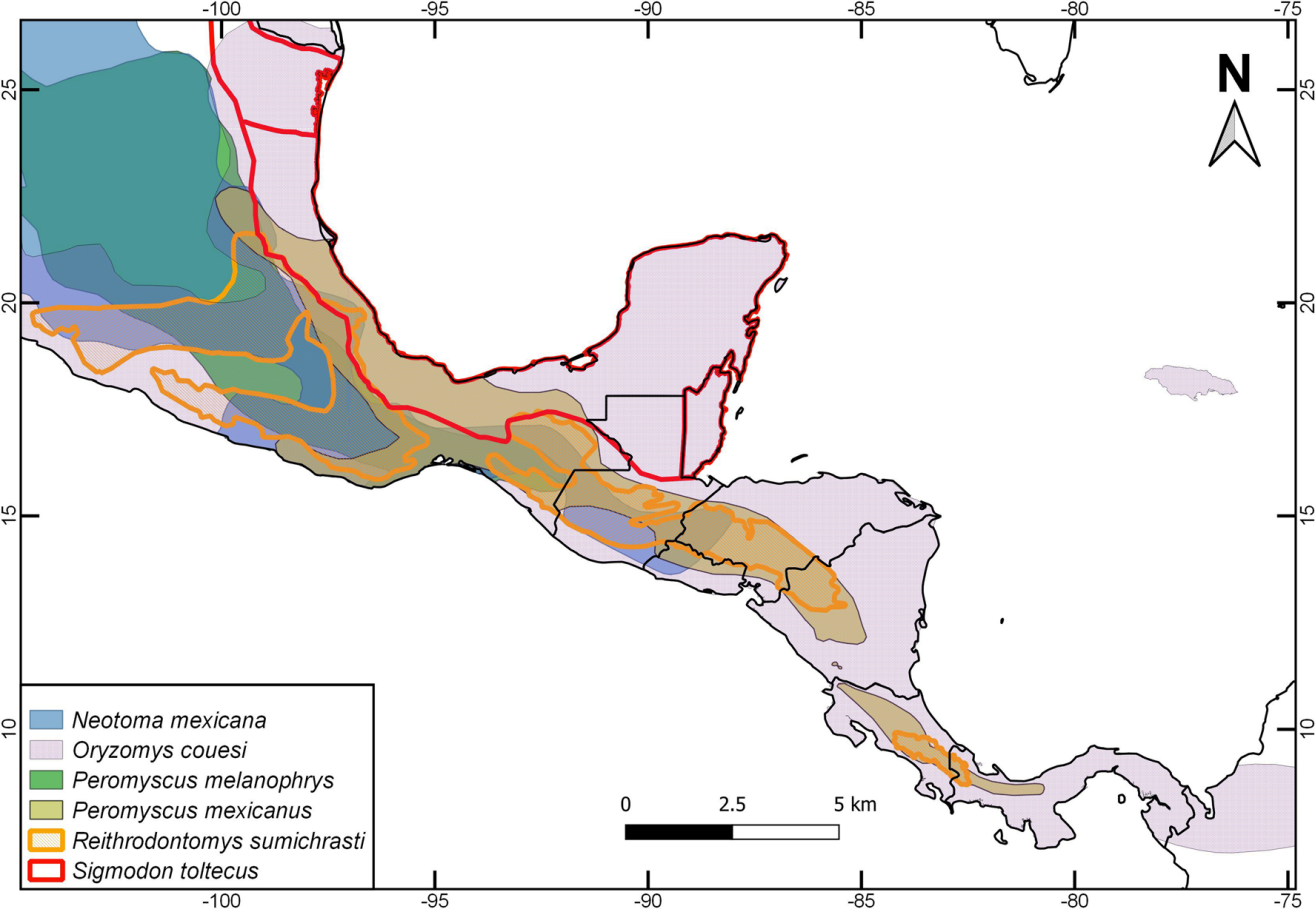
Geographic distributions of rodent reservoir species of mammarenaviruses in Mesoamerica (south of Mexico, Guatemala, Honduras, El Salvador, Nicaragua, Costa Rica and Panama).

Regarding rodent reservoirs, new mammarenaviruses have been isolated and described in 27 of 47 rodent reservoirs; 26 of which are from the Cricetidae family and one is from Muridae, which is a non-native rodent (Table 1). The remaining 20 rodents have been identified as having positive antibodies to some *Mammarenavirus* genotypes, but these viruses have not been isolated (Table 2). The 26 Cricetidae rodents reservoirs (where mammarenaviuses have been isolated) are widely distributed in the Americas, according to IUCN (IUCN 2020); eight of them are found in North America, and the remaining eighteen in Central and South America, from Panama to Chubut province, in Argentina [Insert Fig. 1a–d here]. Also, in smaller regions of the continent—like Mesoamerica (which includes the center of Mexico to Panama)—at least five other rodent reservoir species of mammarenaviruses are overlapping their distributions: *Neotoma mexicana, Oryzomys couesi, Peromyscus melanophrys, Reithrodontomys sumichrasti,* and *Sigmodon toltecus*, all of which are sympatric with *P. mexicanus* (Fig. 2).

**Table 2:** Rodent species bearing antibodies to genotypes of *Mammarenavirus* in the Americas.

Rodent species	*Antibodies to*	Diseases reported	Distribution of rodent reservoir species	Reference
*Baiomys taylori*	WWAV & AMAV	NR	US & MEX	Milazzo et al. (2010)
*Megadontomys nelsoni*	WWAV & AMAV	NR	MEX	Milazzo et al. (2010)
*Neotoma fuscipes*	WWAV, AMAV, TAMV & PICV	NR	US & MEX	Bennett et al. (2000)
*N. lepida*	WWAV, AMAV, TAMV & PICV	NR	US	Bennett et al. (2000)
*N. leucodon*	WWAV & AMAV	NR	US & MEX	Milazzo et al. (2010)
*N. mexicana*	WWAV & AMAV	NR	US, MEX, HON, GUA & SAL	Milazzo et al. (2010)
*N. micropus*	WWAV & AMAV	NR	US & MEX	Milazzo et al. (2010)
*Onychomys leucogaster*	WWAV & AMAV	NR	CAN, US & MEX	Milazzo et al. (2010)
*Oryzomys couesi*	WWAV & AMAV	NR	US, MEX, BEL, COL, CRI, SAL, GUA, HON, NIC & PAN	Milazzo et al. (2010)
*O. palustris*	WWAV & AMAV	NR	US	Milazzo et al. (2010)
*Peromyscus attwateri*	WWAV & AMAV	NR	US	Milazzo et al. (2010)
*P. boylii*	WWAV & AMAV	NR	US & MEX	Milazzo et al. (2010)
*P. californicus*	WWAV, AMAV, TAMV & PICV	NR	US & MEX	Bennett et al. (2000)
*P. eremicus*	WWAV, AMAV, TAMV & PICV	NR	US & MEX	Bennett et al. (2000)
*P. leucopus*	WWAV & AMAV	NR	CAN, US & MEX	Milazzo et al. (2010)
*P. maniculatus*	WWAV, AMAV, TAMV & PICV	NR	CAN, US & MEX	(Bennett et al. 2000 Milazzo et al. 2010)
*P. megalops*	WWAV & AMAV	NR	MEX	Milazzo et al. (2010)
*P. melanophrys*	WWAV & AMAV	NR	MEX	Milazzo et al. (2010)
*P. melanotis*	WWAV & AMAV	NR	US & MEX	Milazzo et al. (2010)
*P. mexicanus*	WWAV & AMAV	NR	MEX, GUA, HON, SAL, CRI, NIC & PAN	Milazzo et al. (2010)
*Reithrodontomys megalotis*	WWAV, AMAV, TAMV & PICV	NR	US & MEX	Bennett et al. (2000)
*R. sumichrasti*	WWAV & AMAV	NR	MEX, GUA, HON, SAL, CRI, NIC & PAN	Milazzo et al. (2010)
*Sigmodon hispidus*	WWAV & AMAV	NR	US	Milazzo et al. (2010)
*S. toltecus*	WWAV & AMAV	NR	MEX & GUA	Milazzo et al. (2010)
*Zygodontomys brevicauda*	PICV	NR	BRA, COL, CRI, FGU, GUY, PAN, SUR, TRT, VEN & BOL	Mattar et al. (2011)

*WWAV* Whitewater Arroyo virus, *AMAV* Amaparí virus, *TAMV* Tamiami virus and *PICV* Pichindé virus; *NR* not reported; *ARG* Argentina, *BEL* Belize, *BOL* Bolivia, *BRA* Brazil, *CAN* Canada, *COL* Colombia, *CRI* Costa Rica, *FGU* French Guiana *GUA* Guatemala, *GUY* Guyana, *HON* Honduras, *MEX* Mexico, NIC Nicaragua, *PAN* Panama, *SAL* El Salvador, *SUR* Suriname, *TRI* Trinidad, *US* United States and *VEN* Venezuela. Published reports of antibodies detected through ELISA tests, according each author’s criteria:

^*^Milazzo et al. (2010): A sample was considered positive if the AOD at 1:80 was > 0.200, the AOD at 1:320 was > 0.200, and the sum of the AOD for the series of fourfold dilutions (from 1:80 through 1:5120) was > 0.750.

^*^Bennett et al. (2000): A serum was considered to be positive to a test antigen if the OD_adjusted_ at 1:80 and the OD_adjusted_ at 1:320 both were ≥ 0.200, and the sum of the OD_adjusted_ for the series of fourfold dilutions (from 1:80 through 1:5,120) was ≥ 0.750.

**Figure 3:**
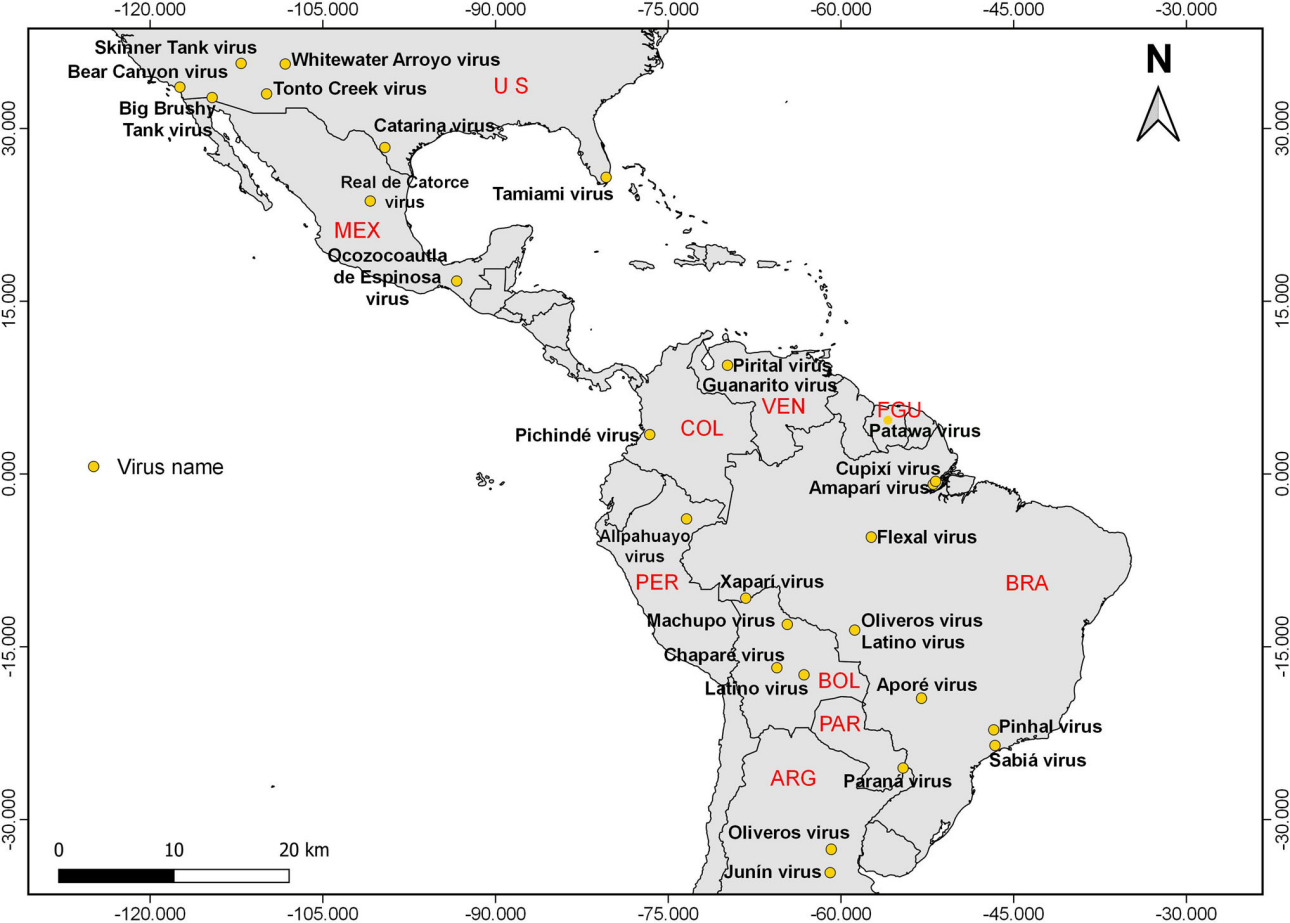
Geographic locations of *Mammarenavirus* genotypes in the Americas. Abbreviations: ARG Argentina, BOL Bolivia, BRA Brazil, COL Colombia, FGU French Guiana, MEX Mexico, PAR Paraguay, PER Peru, US United States, VEN Venezuela.

Considering the genotypes of *Mammarenavirus,* the majority of them have been reported in South America than in North America, i.e. 19 versus. 9 [Insert Fig. 3 here], and nine of those found in South America have been reported in Brazil (Fernandes et al. 2019).

**Figure 4:**
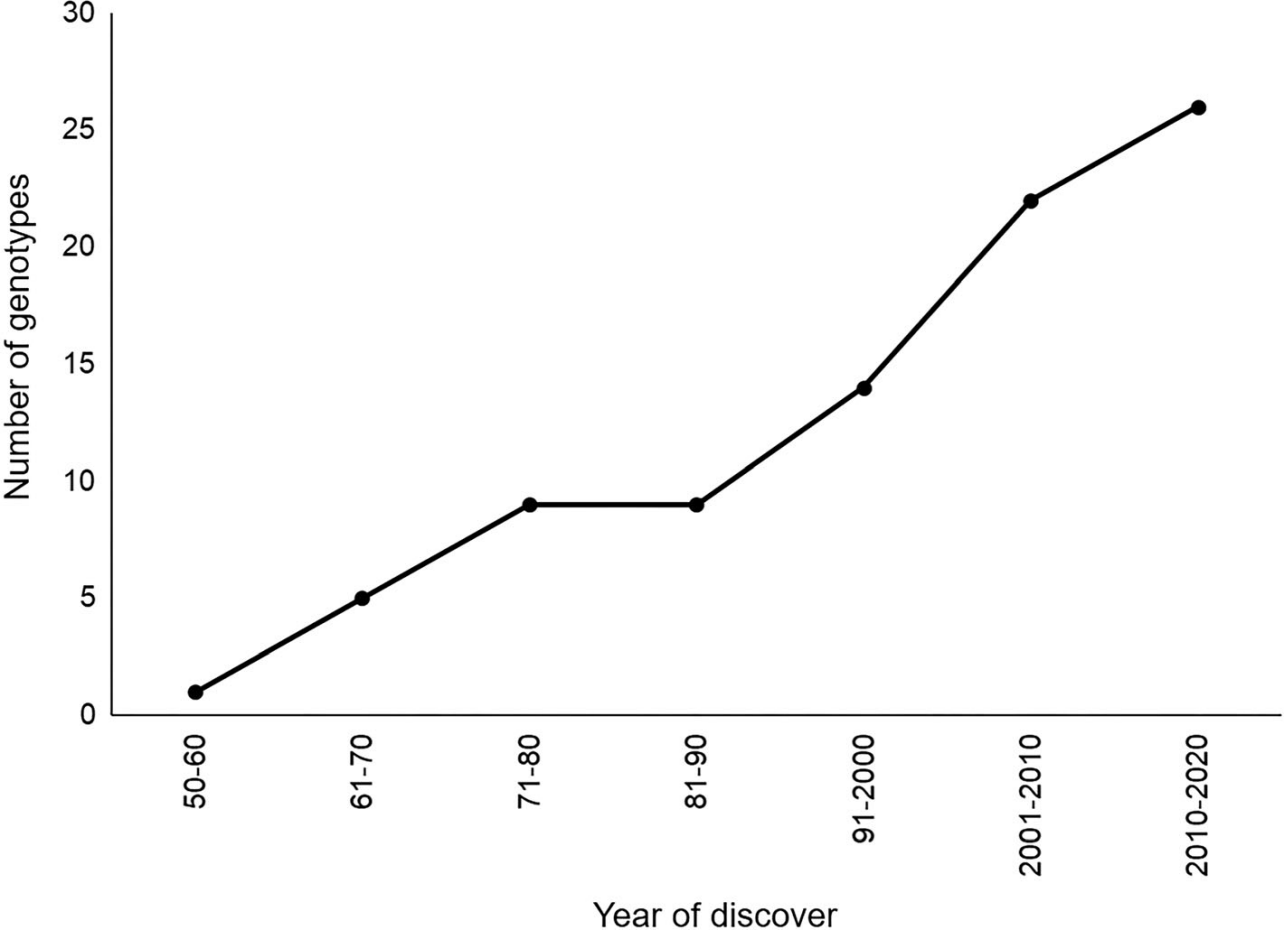
Cumulative number of genotypes registered in the Americas since 1960.

**Figure 5:**
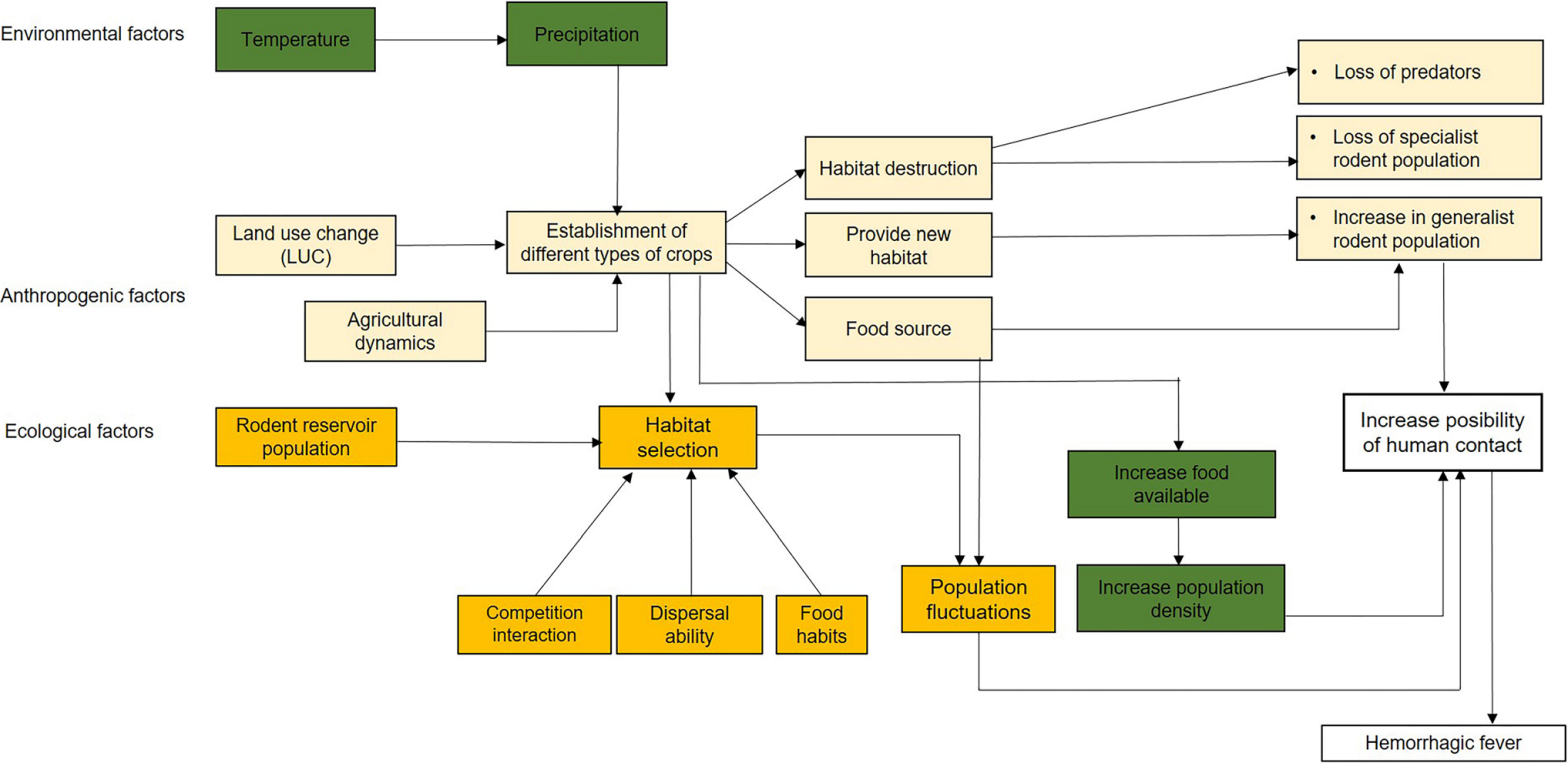
Conceptual model of ecology of rodent reservoirs of mammarenaviruses and their relationship with conservation of virus and transmission of it.

Contrary to prior considerations, a given rodent species may carry more than one virus species (Irwin et al. 2012), as it occurs with the rodent *Neotoma albigula*, which is a reservoir for Whitewater Arroyo virus (WWAV), Big Brushy Tank virus (BBTV), and Tonto Creek virus (TTCV) in the USA (Table 1) (Fulhorst et al. 1996; Milazzo et al. 2008). The same occurs with *Calomys callosus*, which is a reservoir for Machupo virus (MACV) and Latino virus (LATV) in South America (Table 1) (Johnson et al. 1963; Webb et al. 1973). These findings have been markedly fostered by new molecular and serum detection techniques for mammarenaviruses contributing to the discovery and knowledge of these viruses and their reservoirs. In the Americas, from the earliest report, in 1960, until the 1990´s, 12 genotypes had been described (Fig. 4), however, this number almost tripled in the following two decades. In the past 10 years, four new mammarenaviruses have been described in Brazil (Fernandes et al. 2019) and two in Mexico (Inizan et al. 2010; Cajimat et al. 2012).

All the mammarenaviruses cited here are considered exclusive to the Americas, except for the *Lymphocytic choriomeningitis mammarenavirus* (LCMV) (Table 1)*,* an Old World mammarenavirus, considered the prototype of the Arenaviridae family. It is worldwide distributed due to its rodent reservoir: *Mus musculus*, a non-native and also worldwide distributed rodent (Albariño et al. 2010). LCMV in the Americas has been reported in US, Colombia and Argentina (Riera et al. 2005; Foster et al. 2006; Lavergne et al. 2016; Castellar et al. 2017). It can infect other rodent species members of Muridae family in the Old World and can cause disease in humans, which could be asymptomatic or severe (Riera et al. 2005).

Although, epidemic outbreaks and isolated deaths due to VHF from mammarenaviruses have been reported since the 1960s, their reservoir rodents have not been identified in all cases;—for example, those in the USA which have been attributed to WWAV (Byrd et al. 2000; Enserink 2000); those in Brazil, attributed to Flexal virus (FLEV) and Sabiá virus (SABV) (Barry et al. 1994; de Mello Malta et al. 2020); and those in Bolivia, attributed to Chaparé virus (CHAPV) (Escalera-Antezana et al. 2020).

### Land use Change, Reservoir Rodent Ecology, and Hemorrhagic Fever Emergence

According to the information collected, mainly from the 70 s to the 90 s, the factors influencing the emergence of VHFs in the continent are divided into anthropogenic, ecological and environmental, which are interconnected (Fig. 5).

### Anthropogenic Factors

The main anthropogenic factor is land use change (LUC). The indiscriminate establishment of crops in the Americas shapes an agricultural landscape that changes the community structure and population dynamics of the original rodent species, which inhabited the area prior to the establishment of the agricultural landscape. Crops drive the loss of predators and specialist rodents and contribute to an increase in generalist species (Crespo 1966; de Villafañe et al. 1977; Kravetz et al. 1986; Carballal et al. 1988). The loss of some species is due—in part—to the fact that the structure of the rodent habitat is negatively affected as burrows are destroyed. Some individuals are killed, insects are eliminated and the availability of shelters is restricted (de Villafañe et al. 1977). On the other hand, it favors the disappearance of some predators (Kravetz et al. 1986), but also the arrival of others, e.g. dogs, cats or other larger rodents.

Once the agricultural landscape has been established, rodent populations will share that space following the local agricultural dynamics, generating a pulsation-like pattern of rodents’ presence. That is, when a plot is in preparation for sowing, one species will occupy it, while, when it is planted there will be another species, and, yet another one, at harvest time (de Villafañe et al. 1977). A similar pattern has been described for *Sigmodon alstoni* and *Zygodontomys brevicauda* in Venezuela, with species associated with the Venezuelan Hemorrhagic Fever (VHF) (Utrera and Duno 2007). Each rodent species utilizes the agricultural landscape during certain growth-stages of a crop according to its needs for food and protection. Because of this, certain species prove to be more abundant than others, potentially causing outbreaks of VHF (Ellis et al. 1997). In turn, VHF is related to seasonal population fluctuations and periods of higher rodent population density.

### Ecological Factors

Rodent populations that remain in this agricultural landscape are distributed according to their habitat and food preferences. For example, in Pergamino, Argentina, the species involved in the transmission of the Junin virus (*Akodon azarae, Calomys musculinus* and *C. laucha*) occupy agricultural plots differentially (de Villañafe et al., 1977), according to their eating habits, (i.e. if rodent eat grains or grasses). Therefore, the populations of *C. musculinus* and *C. laucha* occupy the cornplots. While *Akodon azarae*, and *Necromys obscurus* occupy those of soybeans or alfalfa (de Villafañe et al. 1977; Kravetz et al. al. 1986; Mills et al. 1991). Additionally, these preferences are reinforced by interspecific competition between *Akodon azarae* and *C. musculinus*, which ends up displacing and confining the former to the edges of agricultural plots where the vegetation cover is more diverse and abundant (Carballal et al. 1988). This type of interaction has not been recorded between *Calomys musculinus* and *C. laucha* (de Villafañe et al. 1977), suggesting their coexistence in agricultural plots. This should favor their role in the transmission of the Junin virus to rural workers (Mills et al. 1991). The occupation of the species—in these habitats— is a direct function of the dispersal ability and the capacity of the rodent species to reproduce, since they are R strategists (i.e., having numerous litters in short periods of time) (de Villafañe et al. 1977).

A relationship has been suggested between density of reservoir rodents (in those cases *Calomys musculinus*) and incidence of disease (Crespo 1966; Mills et al. 1992). For example, Mills et al. (1991), Crespo (1966) and de Villafañe et al. (1977) reported that the population density of *Calomys musculinus* is higher from spring to early southern autumn (between September and March), while *Akodon azarae* is not very abundant at that time and increases its population density between March and April, after harvest.

### Environmental Factors

A bottom-up effect has been suggested causing increased population sizes of reservoir rodents due to changes in precipitation; thus, leading to increased food availability and, in turn, numerous cases of VHF (Mills et al. 1992). Changes in temperature have also been associated to resource availability. For example, Mills et al. (1992) associated low temperatures to low resource availability, because of decreased rain. In turn, sites with temperate climates, less temperature variations and more rain accommodate a larger diversity of rodents, as well higher abundance of *Calomys musculinus* (Mills and Childs 1998; Chiappero et al. 2018).

Other factors influencing spatial patterns and rates of dispersion of VHF in a given territory include the genetics of the rodent populations involved, geographic boundaries, local extinctions of rodents or viruses, environmental variables, and intrinsic properties of a reservoir community which allow it to support long-term maintenance of a virus (Polop et al. 2008). According to Polop et al. (2008), the prevalence of Junin virus in Argentinian rodents could be greater in the area of endemism than in sites far from it, under certain conditions. In the endemic area, reservoir rodent populations are large and genetic flow occurs among them. These areas may favor the presence of generalist rodents given the abundance of food (Polop et al. 2007). However, outside the endemic area reservoir rodent populations are separated from each other, and little or no genetic flow occurs among them; thus, these areas act as islands (Delgado et al. 2008). Environmental conditions and local vegetation determine which rodent populations makes use of these “islands”, as well as when and where. While viruses may infect a local population, if such population does not become abundant after a certain time period, the infectious cycle may be stopped. Nevertheless, this hypothesis requires further study.

### Transmission of mammarenaviruses

The way in which mammarenaviruses keep circulating in the population of reservoir rodents is key in determining the preservation of the virus, and its transmission to other members of the population, to other populations and to humans. In rodent populations there are two routes of transmission. The first of them—the horizontal route—occurs through the aerosolized particles secreted through feces, urine, saliva or sexual route (Sabattini et al. 1977). It has been suggested that the infection also occurs by rodent-rodent contact, specifically among adult male individuals exhibiting aggressive behaviors; thus, causing wounds through which the pathogen is transmitted (Mills et al. 1992). This can increase prevalence of infection in dense populations, since, the prevalence of infection is density-dependent and this, in turn, is associated to seasonal changes. The other route is vertical, which occurs from parent to progeny. It is known that—at least in the case of the Junin virus—the offspring becomes infected after being born, so it is believed that infection occurs via breast milk (Pinheiro et al. 1977; Sabattini et al. 1977). According to Pinheiro et al. (1977) and Vitullo and Merani (1988), the vertical path does not play an important role in the transmission of *Mammarenavirus* under normal circumstances; however, it could be important for the maintenance of the virus in the population in the long term.

Two main transmission routes between rodents and humans have been described for mammarenaviruses: i) through aerosolized particles secreted by rodents in feces, urine, or saliva (Charrel et al. 2001), and ii) through direct contact with a rodent´s body fluids, such as saliva, mucous, or blood through bites from carriers or upon consuming a rodent (Ter Meulen et al. 1996); as it is customary, for example, in some parts of Mexico (Barragán et al. 2007).

### Phylogenetic Studies of Mammarenaviruses and Rodents

The Arenaviridae family is divided into two groups—or “complexes”—based on their antigenic properties: i) the Lassa-Lymphocytic Choriomeningitis complex, present in the Old World, and ii) the Tacaribe complex, in the New World (Queen et al. 2015). These complexes are also differentiated based on the family of rodents carrying them: Muridae in the Old World and Cricetidae in the New World (Arata and Gratz 1975).

The Tacaribe complex is divided into three lineages: A, B, and C. Lineage A is the oldest, containing the viruses FLEV, PARV, PICV, and TAMV. Lineage B contains AMAV, GUAV, JUNV, MACV, SAB, and TCRV and includes all pathogenic viruses except Tacaribe (Bowen et al. 1996). Lineage C contains LATV and OLVV.

Regarding the phylogeny of these viruses, until 2018, researchers have suggested that, since the time of origin of rodents, these viruses evolved in parallel with their reservoirs (Zapata and Salvato 2013; Shi et al. 2018). The first contact between a viral prototype of the Arenaviridae family and a rodent likely occurred in Asia 23 million years ago with rodents from the Muridae family (González et al. 2007). They expanded from Asia to Africa and Europe and, 20 million years ago, they crossed the Bering Strait to North America; where they diverged into the ancestors of the Neotominae and Sigmodontinae subfamilies. The latter probably already carried the ancestors of the mammarenaviruses of the Americas (González et al. 2007; Zapata and Salvato 2013). Researchers previously thought that each viral species was associated with a single rodent species and therefore the rodent´s distribution determined that of the virus (Charrel et al. 2001).

However, based on the complete phylogeny of the Cricetidae rodent family and the currently-known mammarenaviruses in the Americas, it has been suggested that the supposed parallel relationship is rather a co-divergence (Irwin et al. 2012). Mammarenaviruses of the Americas are randomly distributed within the phylogenetic tree of Cricetidae rodents, and therefore a randomly distributed group of pathogenic viruses could infect a variety of rodent species and even other orders of mammals (host-switching) (Irwin et al. 2012). Over time, some *Mammarenavirus* genotypes have been found in more than one rodent species; for example, BCNV infects both *Neotoma microtis* and *Peromyscus californicus* (Irwin et al. 2012). Similarly, LATV, originally found in *Calomys callosus* in Bolivia, was recently detected in *C. callidus* in Brazil (Fernandes et al. 2018).

Still, some authors suggest that the probability of a virus successfully colonizing a new reservoir species is greater if the new species is a close relative of the primary reservoir (Cuthill and Charleston 2013). Therefore, in the evolutionary history of vertebrate RNA viruses, spillovers in reservoirs may have been more common than co-divergence; particularly, among reservoirs sharing a given environment (Shi et al. 2018). Thus, the evolution of arenaviruses appears to be the product of a relationship between co-divergence of a virus and its reservoir, on the one hand, and frequent transmission among sympatric rodent species on the other (Geoghegan et al. 2017).

## Discussion

This review reports, to date, the existence of 47 species of reservoir rodents, bearing 26 genotypes of *Mammarenavirus*; seven of which can cause mild to severe hemorrhagic fevers (VHF) in humans. Two of those seven genotypes have not yet been associated with any known rodent. VHFs due to mammarenaviruses have occurred in Argentina, Bolivia, Venezuela and some isolated cases are known in Brazil and the USA. Twenty—out of the 47 species of rodents—have only tested antibody positive to mammarenaviruses, i.e. no novel mammarenaviruses have been isolated from these rodents. In this regard, the potential role of cross-reactivity—a relatively common phenomenon in heterologous viruses—must be recognized. In fact, Brehm et al. (2002) recognized this reactivity and suggested that it improves protection against subsequent viral stimuli, also, improving the pool of memory of T cells; which is why it is common to find reactivity to more than one type of virus in a single species of rodent. Moreover, rodent species where antibodies have been detected are also located in North America and they are mostly generalists, sympatric and with a wide distribution throughout the American Continent.

Regarding the factors related to the emergence of viral hemorrhagic fever (VHF), land use change has been mentioned repeatedly. In particular, the establishment of agricultural plots, which modifies the rodent community structure and abundance of reservoir species (de Villafañe et al. 1977, 1988; Villafañe and Bonaventura 1987; Mills et al. 1992). There are also mentions to an association with temperature and precipitation (Mills et al. 1992; Polop et al. 2008). In this regard, recent studies confirmed the hypothesis that human alterations in ecosystems create favorable habitats for reservoirs of zoonotic pathogens (Mendoza et al. 2020). In the American continent, this topic has been constantly investigated in cases of VHF emergence by *Orthohantavirus* (Suzán et al. 2008; Andreo et al. 2014; Rubio et al. 2014; Carver et al. 2015; Prist et al. 2021). The conclusions of these authors are important because some of the rodent species are reservoirs of both *Orthohantavirus* and *Mammarenavirus*; and the set of factors promoting transmission of viruses to humans are the same (Prist et al. 2021 and this work).

On the other hand, a concomitant factor that should be explored is climate change. Which, in turn, leads to increases and changes in meteorological patterns (Gubler et al., 2001; Greer et al., 2008; Maroli et al., 2018; Tian and Stenseth, 2019). Climate and meteorological changes have often been studied in the Americas during outbreaks of *Orthohantavirus-associated* pulmonary syndrome (HPS), which has been associated with increases in temperature (Prist et al. 2016), precipitation (Yates et al. 2002), and humidity (EcoHealth Alliance 2019; Everard et al. 2020).

This work has found that—in the continent—certain geographic regions can present between six (Mesoamerica) and up to nine (Brazil, Bolivia, Peru and Panama) species of reservoirs. These areas are consistent with the regions, designated by Han et al. (2015), as areas of high diversity and, on the other hand, areas with high rates of zoonotic risk (García-Peña et al. 2021). These authors project risk scenarios based on anthropogenic factors such as land use, but they also consider the possible risk of exposure of the human population. For this reason, the authors consider it necessary to carry out research in these areas considered as areas prone to zoonotic risk scenarios.

## Conclusion

In the past 60 years, 28 genotypes of *Mammarenavirus* have been described; seven of which are pathogenic to humans. In the past 60 years in the Americas, the number of identified rodent reservoir species of mammarenaviruses has increased from 10 to 47—including specialists and generalists—all of which are common generalist species in peridomestic environments and agricultural areas. The data show that there could be more than one risk spot for the emergency of a new hemorrhagic fever.
